# Mitral valve prolapse associated with celiac artery stenosis: a new ultrasonographic syndrome?

**DOI:** 10.1186/1476-7120-2-28

**Published:** 2004-12-10

**Authors:** Luciano Arcari

**Affiliations:** 1 Guglielmo Marconi University – ASDAC (Updating and Teaching in Cardiology, Scientific Association), ARCA Lazio, Rome, Italy

## Abstract

**Background:**

Celiac artery stenosis (CAS) may be caused by atherosclerotic degeneration or compression exerted by the arched ligament of the diaphragm. Mitral valve prolapse (MVP) is the most common valvular disorder. There are no reports on an association between CAS and MVP.

**Methods:**

1560 (41%) out of 3780 consecutive patients undergoing echocardiographic assessment of MVP, had Doppler sonography of the celiac tract to detect CAS.

**Results:**

CAS was found in 57 (3.7%) subjects (23 males and 34 females) none of whom complained of symptoms related to visceral ischemia. MVP was observed in 47 (82.4%) subjects with and 118 (7.9%) without CAS (p < 0.001). The agreement between MVP and CAS was 39% (95% CI 32–49%). PSV (Peak Systolic Velocity) was the only predictor of CAS in MPV patients (OR 0.24, 95% CI 0.08–0.69) as selected in a multivariate logistic model.

**Conclusion:**

CAS and MVP seem to be significantly associated in patients undergoing consecutive ultrasonographic screening.

## Background

Celiac artery stenosis (CAS) may be caused by atherosclerotic degeneration, as observed in different vascular districts, or by extrinsic compression (ECCA) usually exerted by an abnormally developed arched ligament of the diaphragm [1-7]. ECCA may cause abdominal or epigastric pain triggered by meals (angina abdominis), a syndrome called Celiac Artery Compression Syndrome (CACS) [1,2]. Resolution of symptoms after surgical resection of the arched ligament has been frequently reported. However, some aspects, such as the vascular etiology of symptoms, are still controversial, as the improvements observed after surgery may be attributed to the concurrent destruction of the celiac ganglion rather than to the severance of the arched ligament.

Mitral Valve Prolapse (MVP) is the most common valvular disorder, with a prevalence of about 6–7% in the general population [8,9]. It consists of systolic displacement of one or both valvular leaflets into the left atrium, with or without mitral regurgitation. Patients with MVP may also be affected by other cardiac anomalies, such as prolapse of the aortic valve and/or of the tricuspid valve, ostium secundum interatrial sect defect, atrio-ventricular left accesses, and/or extra-cardiac anomalies, such as pectus excavatum and loss of the physiological curvature of the thoracic spine, with greater frequency with respect to the general population. An increased incidence of MVP has been demonstrated in connective tissue disorders, especially in Marfan's syndrome [10]. Particular genic anomalies have been associated with MVP [11-13].

The association between MVP and CAS has not been extensively investigated. Accordingly, this study was aimed at verifying this hypothesis based on a possible common origin of the two conditions, i.e. the abnormal development of connective tissue causing both ECCA exerted by the arched ligament of the diaphragm and exuberance of valvular mitral tissue.

## Methods

### Study population

The study population consisted of 1560 out of 3780 (41%) consecutive patients undergoing echocardiographic assessment of MVP, who also had celiac artery Doppler ultrasound performed between November 1999 to September 2004.

### Echocardiographic examination

MVP was defined as clear-cut billowing of one or both mitral leaflets across the mitral annular plane in 2-dimensional parasternal long axis recording or >2 mm late systolic posterior displacement of mitral leaflets by M-mode. A >4 mm displacement defined a moderate-severe prolapse.

**Figure 1:**
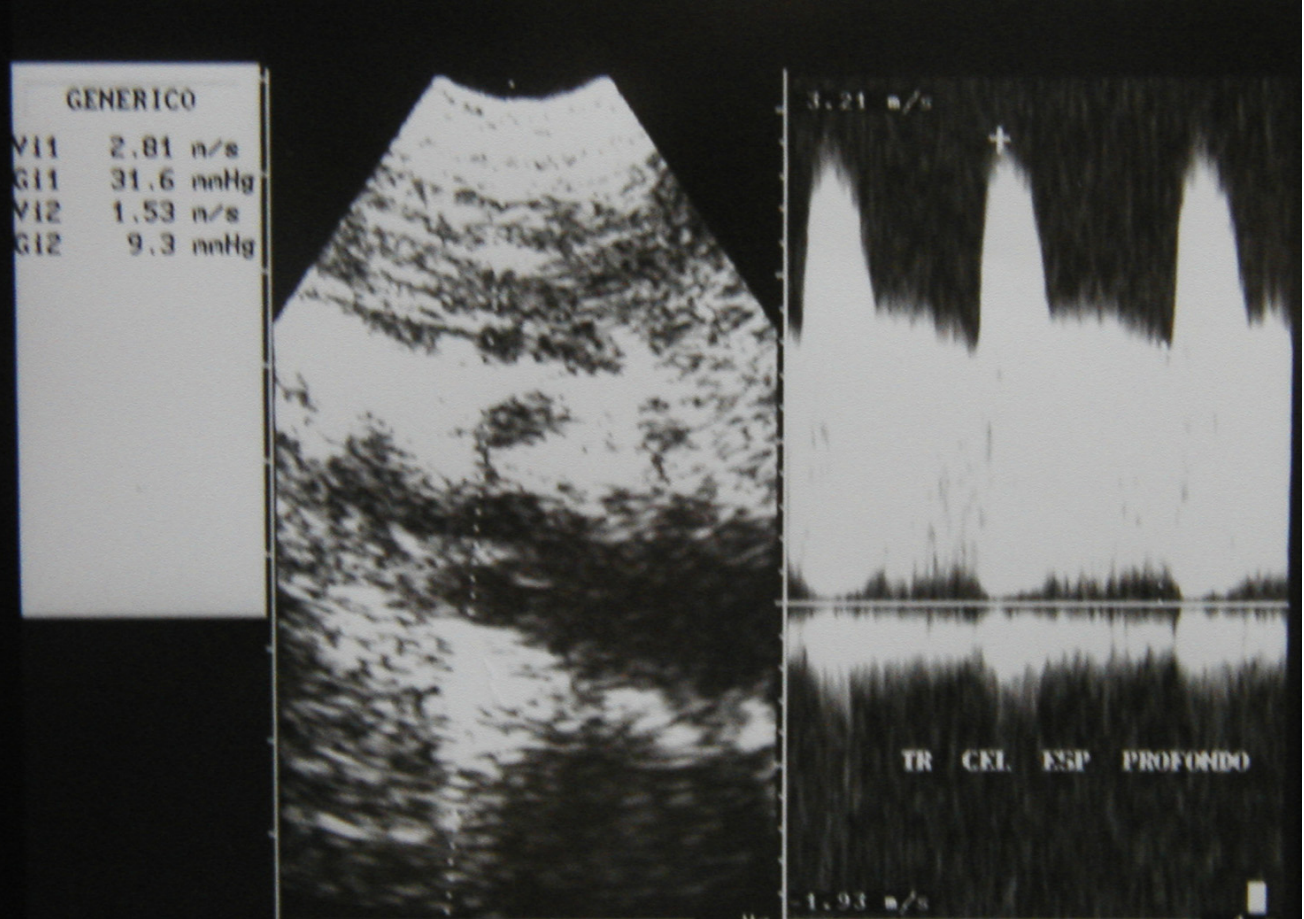
Continuous Doppler of Celiac Artery Stenosis, deep expiration.

**Figure 2:**
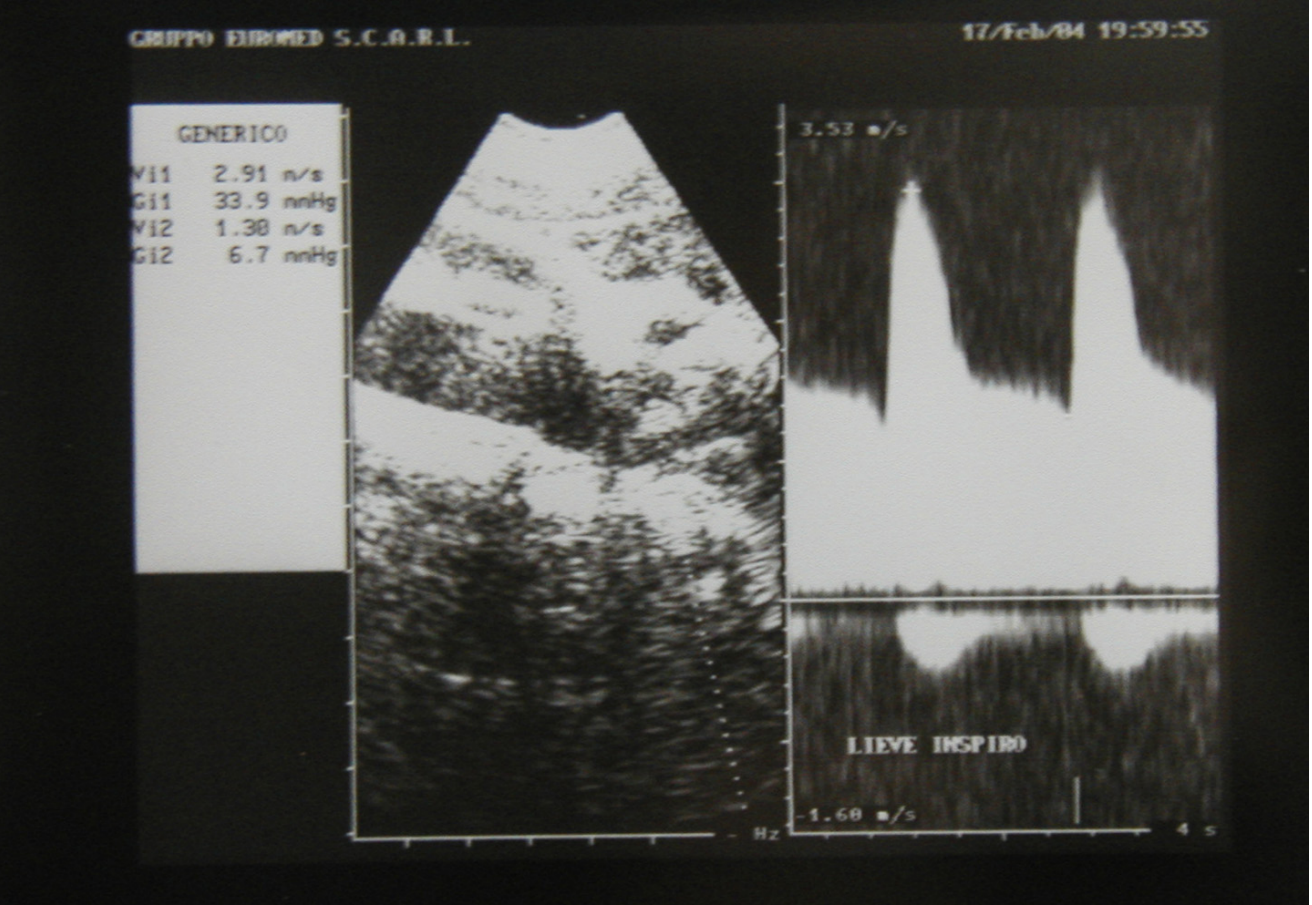
Continuous Doppler of Celiac Artery Stenosis, modeste inspiration.

Ultrasonography (HP Sonos 1000, Tohsiba Corevision, Esaote Caris, Esaote Caris plus, Kontron Iris) was performed without intestinal preparation to limit meteorism. The digestive phase was taken into account, because it may influence visceral arterial flow. The evaluation of arterial flow in the celiac tract was recorded by continuous Doppler (CW) and, whenever possible, with the aid of the color-Doppler signal. No corrections of flow velocity by evaluation of the cosine of the insonorisation axis and the axis of the vessel were used: in this way, flow velocity can never be overestimated. The Doppler signal was collected in apnea during inspiration. During expiration, an increase in the compression of the celiac tripod by the arched ligament occurs in the cases of CAS caused by ECCA, with concurrent increase in flow velocity with respect to the velocity during inspiration [14]. An example is provided in fig. 1, 2, 3. Figures 4,5,6,7,8,9 are examples of patients with CAS and MVP.

**Figure 3:**
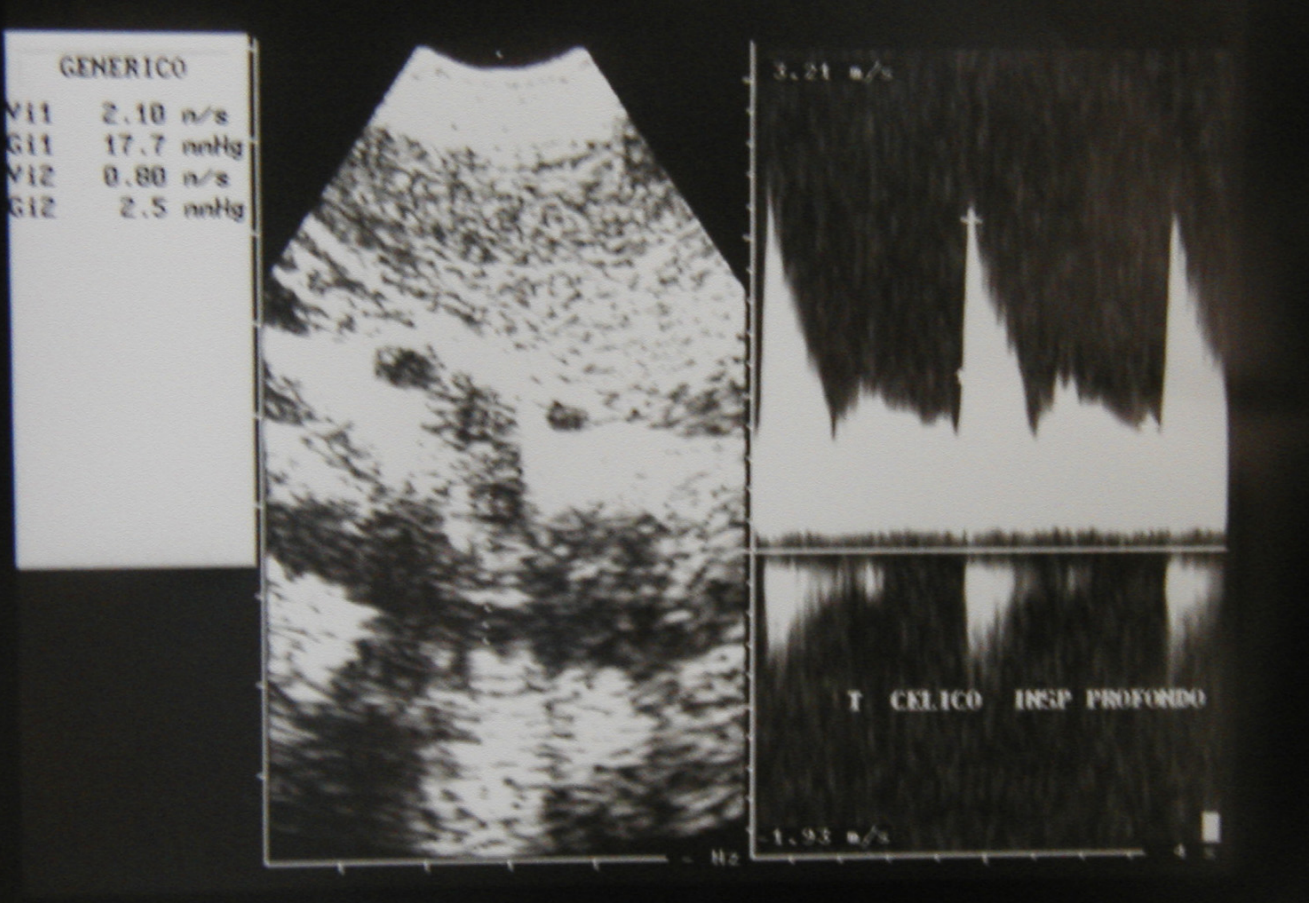
Continuous Doppler of Celiac Artery Stenosis, deep inspiration.

CAS was defined as severe in case of Peak Systolic Velocity (PSV) flow velocity in the upper celiac tract greater than 2.0 metres/second [15,16] and End Diastolic Velocity (EDV) greater than 0.5 metres/second. PSV between 2.0 and 2.5 metres/second defined moderate CAS, whilst those > 2.6 metres/second identified severe CAS.

### Statistical analysis

Continuous variables are expressed as means ± 1 standard deviation (SD). Differences between groups were compared using Student's t-test and chi-square test, as appropriate. Association between CAS and MPV was performed using the Kappa statistics, estimated with 95% confidence interval.

Univariate odds ratio (OR) along with their corresponding 95% confidence intervals were computed for describing association of clinical variables with MPV in CAS patients, using a logistic regression model. Selection of variables significantly associated with MPV in CAS patients was made using a multivariate logistic model. Selection criterion was the Akaike Information Criterion, applied backward to the multivariate logistic model. The statistical significance was settled at a p value < 0.05. The R (release 1.9) statistical package and the Harrell's Design and Hmisc libraries were used for analysis.

## Results

The incidence of MVP in patients with (10.6%) and without (9.3%) assessment of CAS did not differ significantly.

Among those undergoing both examinations, CAS was found in 57/1560 (3.6%) patients. The clinical characteristics of patients with and without CAS are reported in Table 1. The incidence of CAS was significantly higher among subjects with as compared to those without MVP (28% vs. 0.7%, OR 5.11 95% CI 3.43 to 7.61; p < 0.001). Likely, the incidence of MVP was significantly higher among subjects with as compared to those without CAS (82.4% vs 7.9 %, OR 55.11 95% CI 27.17 to 111.97; p < 0.001). Thus, the overall concordance between CAS and MVP was 39% (95% CI 0.28 to 0.49). MPV as indicator of CAS has a high sensitivity (0.82 95% CI 0.71 to 0.90) and specificity (0.92 95% CI 0.91 to 0.92).

**Table 1:** Clinical characteristics of patients with and without CAS.

	**CAS (n= 57)**	**No CAS (n= 1503)**	**P-value**
**Age**	40 ± 21	46 ± 14	0.002
**Male**	23 (40%)	691 (46%)	0.400
**MVP**	47 (82.4%)	118 (7.9%)	<0.001

No patient with CAS was complaining of symptoms related to visceral ischemia, i.e. abdominal or gastric pain in concurrence with food ingestion.

Thirteen (22.8 %) of the 57 subjects with CAS were suffering from cardiovascular disorder. In particular, a macrovascular atherosclerotic disorder (myocardial infarction, carotid plaques, abdominal aorta aneurism, peripheral arterial disease involving the lower limbs) was ascertained in 7 subjects (6 male and 1 female, mean age 71+14 years), whilst no sign of macrovascular atherosclerosis was found in the remaining 50 subjects (16 males and 34 females, mean age 35+12 years).

In patients with CAS, factors associated with MPV are age, sex, PSV and BMI. At multivariate analysis only PSV resulted as an independent factor associated with MPV (OR 0.24 95% CI 0.08 to 0.69).

## Discussion

The incidence of CAS is not clearly established, the majority of the literature consisting of isolated reports [17-21]. Surprisingly, one study [22] evaluating the incidence of CAS in 400 asymptomatic subjects undergoing angiographic examination because of hepatic neoplasm, reported a 7.3% incidence of hemodinamically significant CAS. Its origin ECCA in 55% and atherosclerosis in 10%, whilst it remained not determined in 35% of cases.

The incidence of CAS and MVP in the present study dealing with an unselected population was 3.7% and 10.6%, respectively. In addition, this is the first report, to our knowledge, to demonstrate a strong association between the two conditions.

CAS may be caused by ECCA or atherosclerosis. Unfortunately, the ultrasound technique has not enough resolution to allow an etiological discrimination that may be difficult to get even with angiographic examination [22]. However, clinical information may be of help to hypotesize the etiology of CAS in our population. Indeed, CAS was likely due to atherosclerotic disease in the 7 subjects with macrovascular atherosclerotic disorders, whilst it could be secondary to ECCA in the 50 subjects with no feature of atherosclerosis. Of interest, MVP was found in no subject of the first group, whilst it was present in 47/50 (94%) of the second group. Characteristics of CAS subjects according to the presence of atherosclerotic markers are reported In Table 2. On the basis of these findings, it is logical to hypothesize that ECCA represents the factor explaining the association between CAS and MVP. The two pathological conditions may share a common genetic disorder causing a similar defect of the connective tissue at the two anatomic sites. Nevertheless, no correlation was found between CAS severity and degree of MVP.

**Table 2:** Characteristics of CAS patients according to the presence of atherosclerotic disorders. Univariate OR (95% CI) are computed for the association of each single variable with MPV.

	**N**	**No MPV (N = 10)**	**MPV (N = 47)**	**Combined (N = 57)**	**OR (95% CI)**
**Sex**	57	70% (7)	34% (16)	40% (23)	0.22 (0.005,0.97)
**Age**	57	41.7/66.0/72.7	20.0/33.0/47.5	23.0/36.0/55.0	0.15 (0.04, 0.57)
**PSV**	53	2.6/2.8/3.5	2.1/2.4/2.8	2.2/2.5/2.9	0.24 (0.08, 0.69)
**EDV**	53	0.9/1.0/1.2	0.7/0.85/1.0	0.7/0.9/1.1	0.43 (0.18, 1.05)
**BMI**	48	23.8/27.4/30.3	19.0/21.5/23.0	18.9/21.2/23.2	0.10 (0.01, 0.69)

## Conclusions

The results of this study demonstrate that CAS is a relatively frequent finding among patients undergoing Doppler sonography of the celiac artery and is frequently associated to MVP. On the basis of these findings, further investigations are warranted aimed at determining the exact incidence of ECCA associated with MVP, the family distribution of the association between MVP and ECCA, or the prognostic implication of ECCA in subjects with MVP. Additionally, genetic studies could be advisable in subjects presenting with MVP associated to ECCA.

## List of abbreviations

CAS Celiac Artery Stenosis

MVP Mitral Valve Prolapse

ECCA Extrinsic Compression of Celiac Artery

CACS Celiac Artery Compression Syndrome

PSV Peak Systolic Velocity

EDV End Diastolic Velocity

**Figure 4:**
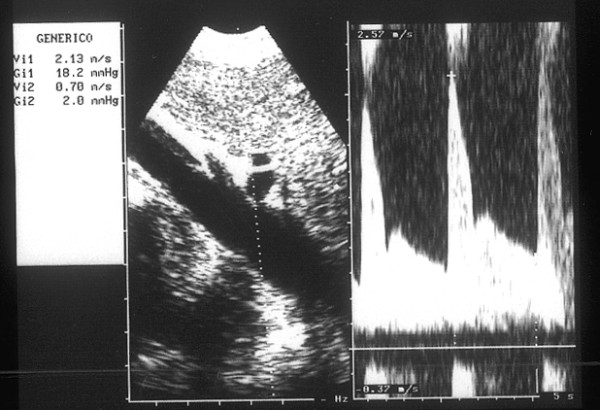
Continuous Doppler of Celiac Artery Stenosis

**Figure 5:**
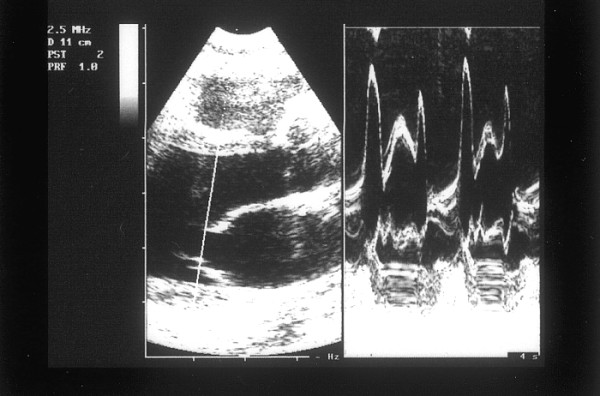
Mitral valve Prolapse, M-mode, patient of fig. 4

**Figure 6:**
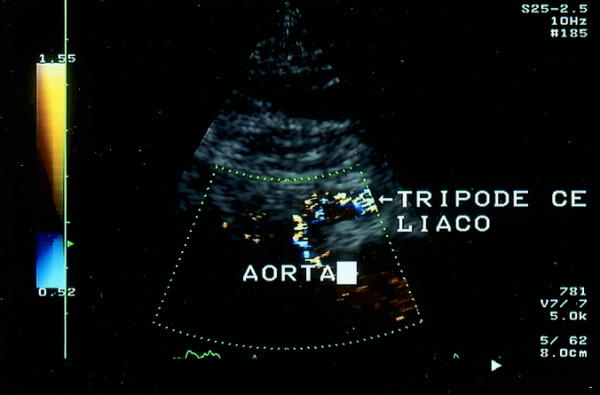
Color Doppler of aorta: celiac artery stenosis (longitudinal proiection)

**Figure 7:**
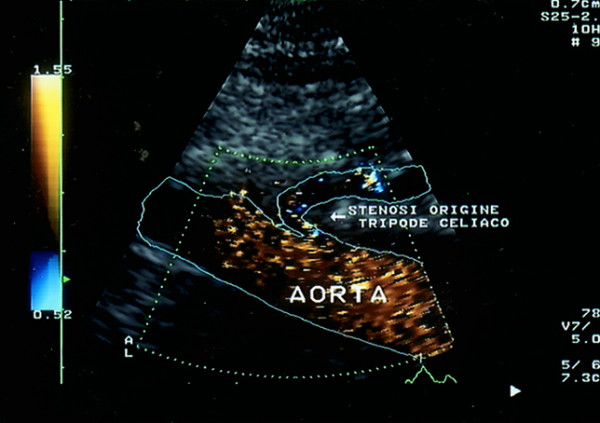
Color Doppler of aorta : celiac artery stenosis (longitudinal proiection)

**Figure 8:**
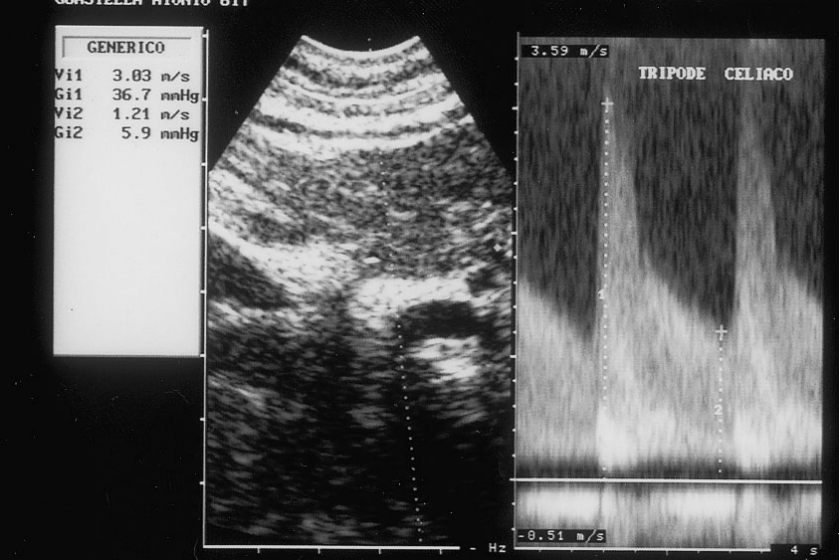
Continuous Doppler of Celiac Artery Stenosis.

**Figure 9:**
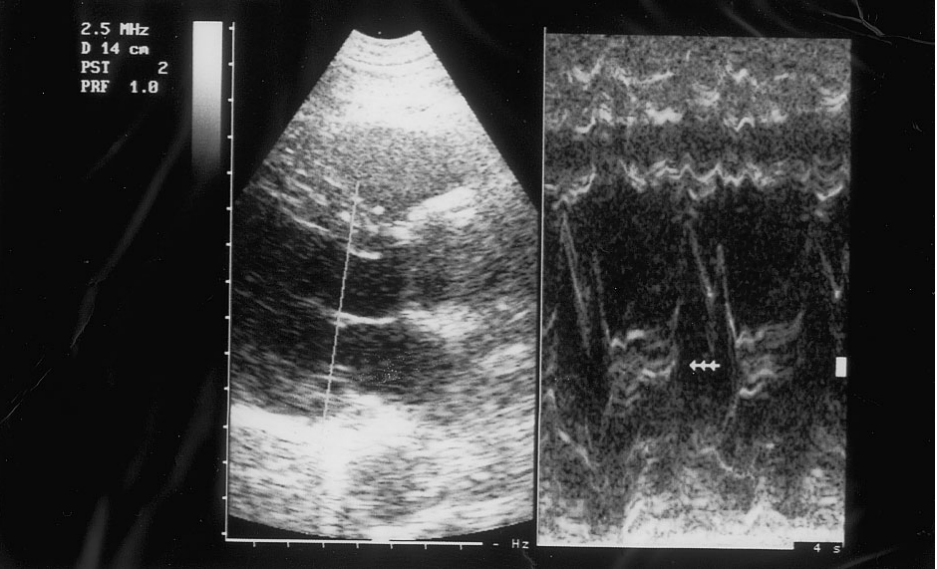
Mitral valve Prolapse, M-mode patient of fig. 8
